# Modulation of glucose metabolism by a natural compound from *Chloranthus japonicus* via activation of AMP-activated protein kinase

**DOI:** 10.1038/s41598-017-00925-y

**Published:** 2017-04-10

**Authors:** Rongkuan Hu, Huan Yan, Xiaoyan Fei, Haiyang Liu, Jiarui Wu

**Affiliations:** 1 Hefei National Laboratory for Physical Sciences at Microscale and School of Life Sciences, University of Science & Technology of China, Hefei, 230027 China; 2 State Key Laboratory of Phytochemistry and Plant Resources in West China, Kunming Institute of Botany, Chinese Academy of Sciences, Kunming, 650201 China; 3 Key Laboratory of Systems Biology, Institute of Biochemistry and Cell Biology, Shanghai Institutes for Biological Sciences, Chinese Academy of Sciences, Shanghai, 200031 China; 4 School of Life Science and Technology, ShanghaiTech University, Shanghai, 201210 China; 5 Department of Biochemistry, University of Texas Southwestern Medical Center, Dallas, TX 75390 USA

## Abstract

AMP-activated protein kinase (AMPK) is a key sensor and regulator of glucose metabolism. Here, we demonstrated that shizukaol F, a natural compound isolated from *Chloranthus japonicus*, can activate AMPK and modulate glucose metabolism both *in vitro* and *in vivo*. Shizukaol F increased glucose uptake in differentiated C2C12 myotubes by stimulating glucose transporter-4 (GLUT-4) membraned translocation. Treatment of primary mouse hepatocytes with shizukaol F decreased the expression of phosphoenolpyruvate carboxykinase 2 (PEPCK), glucose-6-phosphatase (G6Pase) and suppressed hepatic gluconeogenesis. Meanwhile, a single oral dose of shizukaol F reduced gluconeogenesis in C57BL/6 J mice. Further studies indicated that shizukaol F modulates glucose metabolism mainly by AMPKa phosphorylation activity. In addition, we also found that shizukaol F depolarizes the mitochondrial membrane and inhibits respiratory complex I, which may result in AMPK activation. Our results highlight the potential value of shizukaol F as a possible treatment of metabolic syndrome.

## Introduction

AMP-activated kinase (AMPK) is widely recognized as an efficient sensor of intracellular and whole-body energy metabolism. It is a heterotrimeric serine/threonine kinase that regulates fatty acid and glucose homeostasis, and can also be used as an attractive target for the treatment of these related disorders^1^. AMPK appears to be regulated by many tissue derived hormones, including leptin and adiponectin. Leptin activates AMPK to increase fatty acid oxidation in skeletal muscle and hepatocytes, while adiponectin, previously identified as a key regulator of lipid and glucose metabolism, also stimulates AMPK activity in several types of tissues^2^. Meanwhile, AMPK is activated in response to a variety of stresses, including nutrient deprivation, hypoxia, inhibition of the respiratory chain, and the presence of inhibitors of ATP synthases. On the molecular level, Calmodulin-dependent protein kinase kinase (CaMKKb), a protein that plays a role in the calcium/calmodulin-dependent (CaM) kinase cascade, and the tumor suppressor serine/threonine kinase 11 (STK11), also known as liver kinase B1 (LKB1) are two upstream kinases that are known to activate AMPK by phosphorylating AMPKa on Threonine 172^3^.

Under conditions of energy depletion, AMPK is activated and then inhibits ATP consuming pathways, including fatty acid synthesis, cholesterol synthesis and gluconeogenesis. Meanwhile, AMPK stimulates glucose uptake and glucose oxidation to restore overall cellular energy homeostasis^4^. In regulating fatty acid metabolism, AMPK up-regulates the phosphorylation of acetyl-CoA carboxylase (ACC) at Serine 79, resulting in decreased conversion from acetyl-CoA to malonyl-CoA^5^. On the other side, AMPK leads to a decrease of malonyl-CoA levels by regulating the phosphorylation of malonyl-CoA decarboxylase (MCD). Malonyl-CoA further inhibits carnitine-palmitoyl-CoA transferase 1 (CPT1), which is responsible for transporting long-chain fatty acids into mitochondria to be oxidized^6^. For glucose metabolism, AMPK activation suppresses the expression of two key gluconeogenic enzymes, phosphoenolpyruvate carboxykinase 2 (PEPCK) and glucose-6-phosphatase (G6Pase) which in turn inhibits gluconeogenesis^7^. In addition, AMPK increases glucose uptake by stimulating the transfer of GLUT-4 from cytoplasm to membrane^8^. The function of AMPK in regulating glucose metabolism has been demonstrated with several AMPK activators, such as metformin and AICAR, to increase AMPKa phosphorylation and mediates glucose metabolisms both *in vivo* and *in vitro* ^9, 10^. Thiazolidinedione (TZDs) up-regulates the cellular AMP/ATP ratio, which in turn activates AMPK activation and suppresses gluconeogenesis^11^.

Several compounds have been reported to stimulate AMPK activity. For example, Metformin increases AMPKa phosphorylation and mediates glucose metabolism^12^. Rosiglitazone, a clinical drug used to control hyperglycemia, also activates AMPK and regulates PPAR gamma expression^13–15^. Arctigenin activates AMPK via the inhibition of mitochondria complex I and ameliorates metabolic disorders in ob/ob mice^16^. Shizukaol D extracted from traditional Chinese medicine *Chloranthus japonicus* has been confirmed to inhibit the hepatic fatty acid contents by activating AMPK^17^. Shizukaol F and shizukaol D are lindenane-type disesquiterpenoids, which are all isolated from *Chloranthus japonicus* ^18, 19^. Though they have the same skeleton, shizukaol F contains a unique 18-membered macrocyclic trimester ring and a hydroxyl group at C-4′ and shizukaol D has an acetoxyl group attached at C-15′^19, 20^. Besides, shizukaol F exhibits anti-HIV RNase activity and inhibits PMA-induced homotypic aggregation of HL-60 cells^20, 21^. While, there are no reports of shizukaol F modulates metabolism until now. Given the fact that shizukaol D regulate lipid metabolism in hepatic cells, we proposed that shizukaol F may modulate metabolic activity. In this study, our results showed that shizukaol F activated AMPK, increased the glucose uptake in skeletal muscle cells and reduced gluconeogenesis both in primary hepatic cells and *in vivo* via an AMPKa phosphorylation dependent mechanism. Further results showed that the activation of AMPK by shizukaol F is caused by inhibition of mitochondrial complex I activity.

## Results

### Identification of shizukaol F as an AMP-activated protein kinase (AMPK) activator

**Figure 1:**
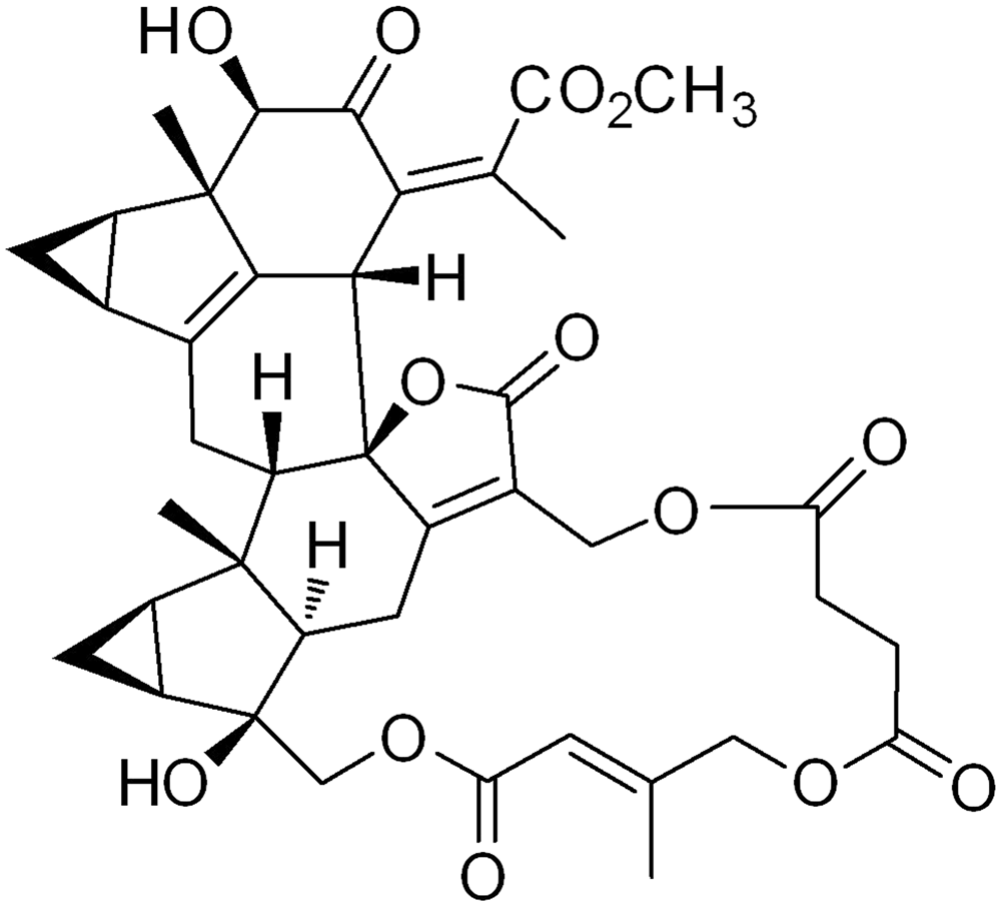
Chemical structure of shizukaol F from *Chloranthus japonicus*.

**Figure 2:**
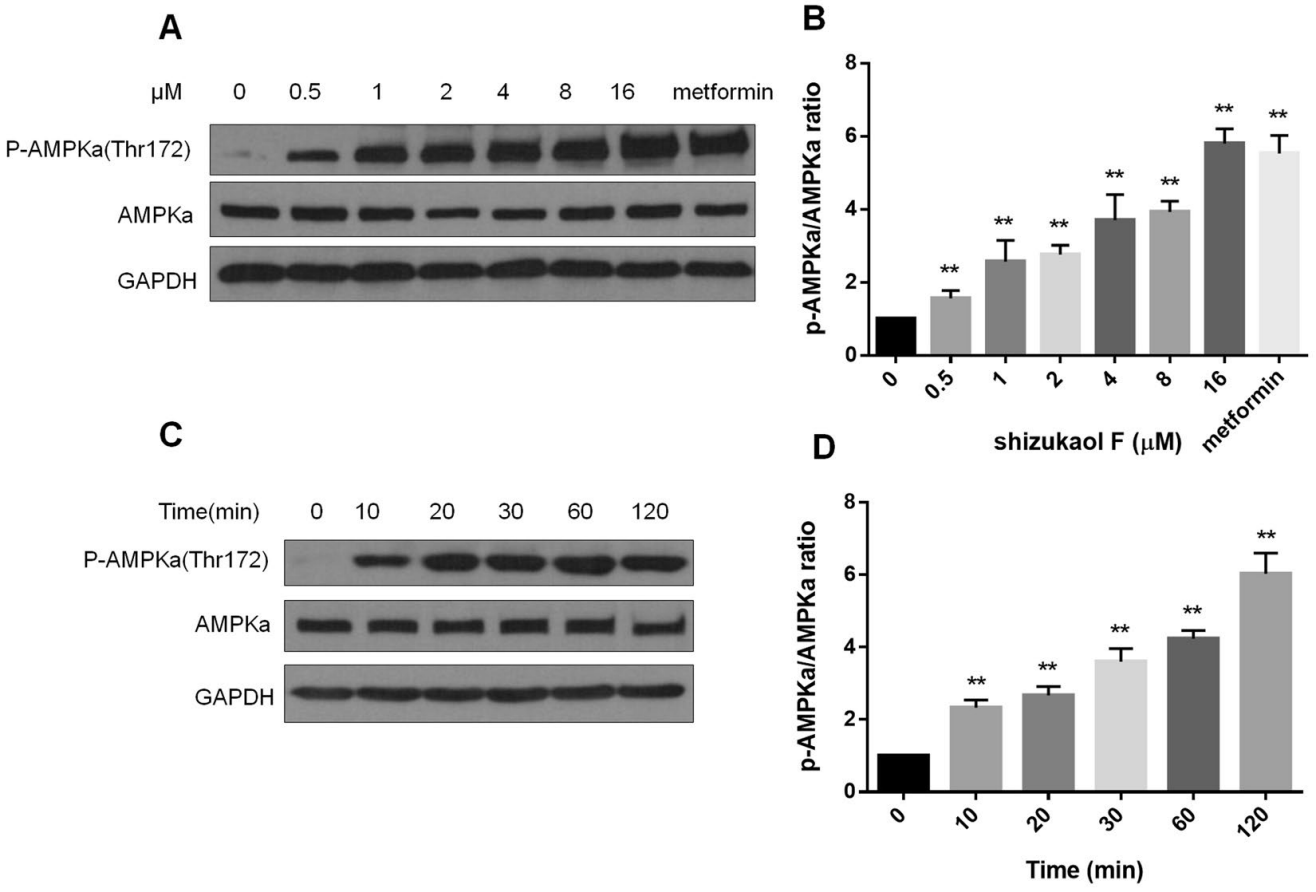
Shizukaol F increases AMPKa phosphorylation in C2C12 cells. Immunoblotting result showed the phosphorylation levels of AMPKa treated with shizukaol F. (**A**) C2C12 cells were treated with shizukaol F at indicated concentrations for 1 h, and 2 mM metformin was used as a positive control. (**C**) The cells were treated with 1 μM shizukaol F for indicated time points. (**B**), (**D**) The phosphorylation levels of AMPKa was quantified from at least three independent experiments. *P < 0.05; **P < 0.01 compared to corresponding controls (one way ANOVA).

Shizukaol F (Fig. 1) was extracted from *Chloranthus japonicus* as previously described^20, 22^. To assess the potential effect of shizukaol F on energy metabolism, we first analyzed the cytotoxicity of shizukaol F in differentiated C2C12 cells. As we observed, treatment of shizukaol F didn’t change the cell viability at various doses for up to 48 hours (Fig. S1A). We then treated C2C12 cells with shizukaol F at the indicated concentrations for 1 h. 2 mM metformin was set as a positive control. The AMPK activity was analyzed by immunoblotting with the specific antibody for phosphorylated AMPKa (Thr 172). As a result, incubation of shizukaol F activated AMPKa phosphorylation in a dose-dependent manner (Fig. 2A,B). In addition, we confirmed the activity of AMPK with 1 μM shizukaol F for different time points (Fig. 2C,D).

### Shizukaol F modulates intracellular glucose metabolism

**Figure 3:**
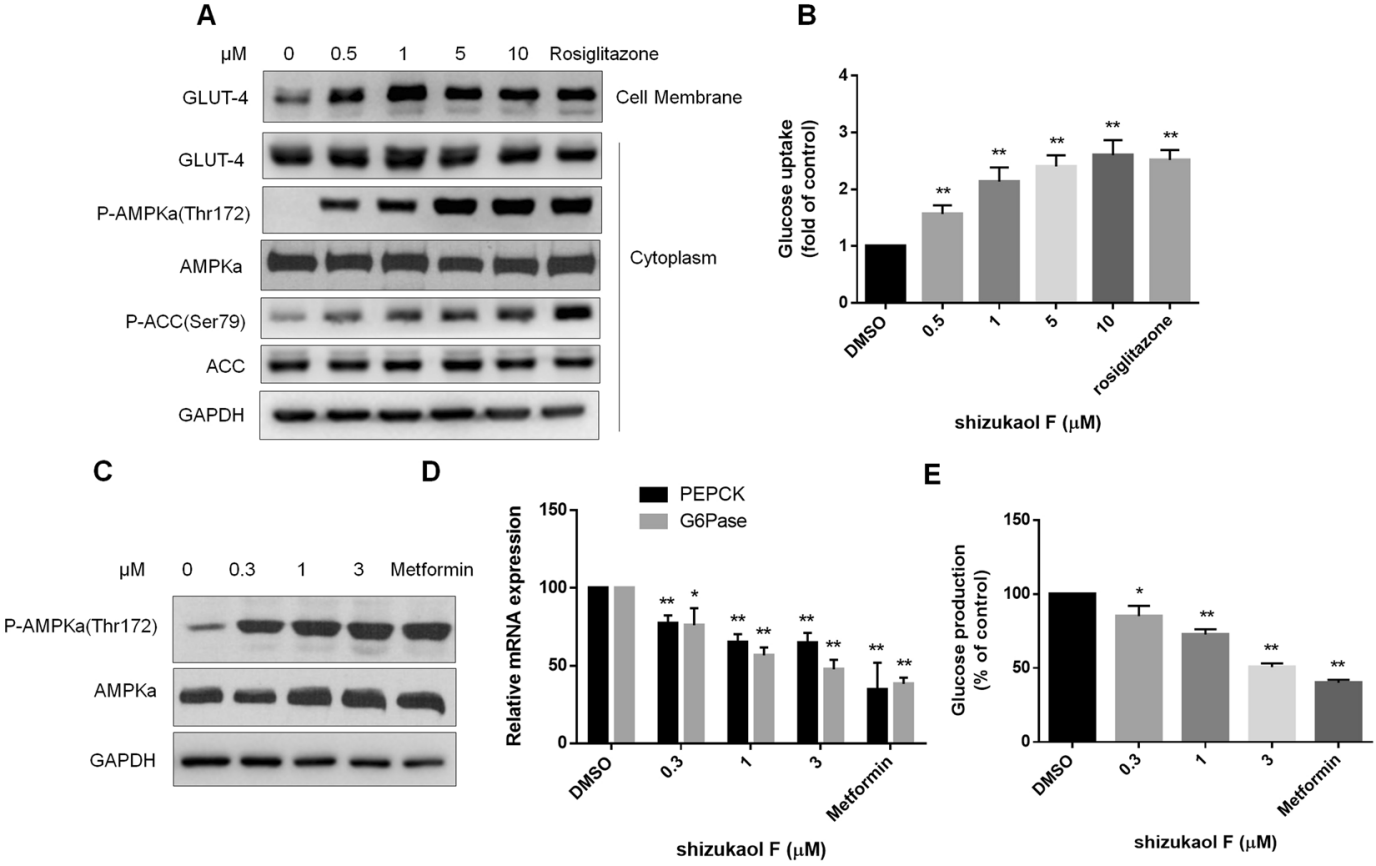
Shizukaol F increases glucose uptake and inhibits gluconeogenesis intracellular. After treatment with the indicated concentrations of shizukaol F for 24 h. (**A**) Shizukaol F increased the phosphorylation of AMPKa and stimulated the GLUT-4 translocation from plasm to membrane. (**B**) The 2-deoxy-D-glucose uptake was measured in C2C12 myotubes after treated with shizukaol F at indicated concentrations for 24 h (n = 3). **P < 0.05 (one way ANOVA). The gluconeogenesis ability in primary mouse hepatic cells was measured after treating with shizukaol F overnight. (**C**) Shizukaol F increased the phosphorylation of AMPKa in primary hepatocytes, and suppressed the PEPCK/G6Pase gene expression (**D**). *P < 0.05; **P < 0.05 (two way ANOVA). n = 3 independent biological replicate experiments. The glucose production in primary hepatocyte was measured after treated with shizukaol F overnight (**E**). **P < 0.05 (one way ANOVA). n = 3 independent biological replicate experiments.

Several studies have shown that the phosphorylation of AMPKa at Thr 172 leads to up-regulated glucose uptake in skeletal muscle cells and decreased gluconeogenesis in hepatic cells^1, 23^. To determine the function of shizukaol F on glucose metabolism, we measured the glucose uptake in a differentiated mouse muscle cell line (Fig. S2), C2C12, after treatment with the indicated concentrations of shizukaol F for 24 h. As shown in Figs 3A and S3, under these conditions, shizukaol F increased phosphorylation of AMPKa (Thr 172) and stimulated the translocation of GLUT-4 from cytoplasm to membrane, which in turn led to an up-regulation of glucose uptake (Fig. 3B).

In addition, we analyzed gluconeogenesis ability in isolated mouse hepatic cells after treatment with shizukaol F overnight. Interestingly, exposure to shizukaol F increased the phosphorylation of AMPKa (Figs 3C and S3D), and suppressed the expression of PEPCK and G6Pase, which were important to gluconeogenesis in hepatocytes^24^ (Fig. 3D). As a result, shizukaol F suppressed gluconeogenesis in primary hepatic cells (Fig. 3E) without changing cell viability (Fig. S1B).

### Shizukaol F inhibits gluconeogenesis and increases AMPKa phosphorylation *in vivo*

**Figure 4:**
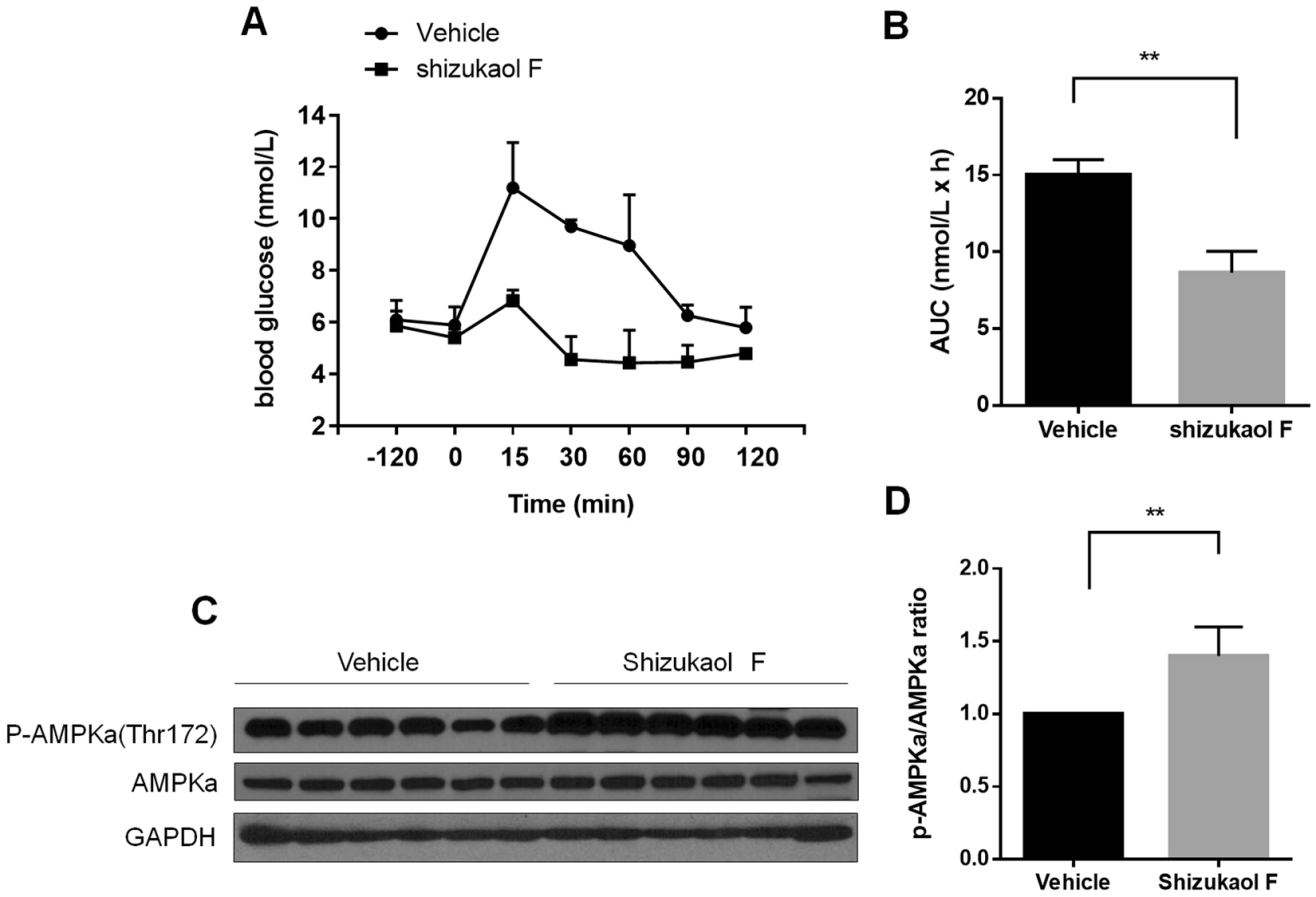
Acute effect of shizukaol F on gluconeogenesis and hepatic AMPKa phosphorylation in C57BL/6 J mice. Mice were treated as described in methods. In the pyruvate tolerance test, blood glucose levels (**A**) were measured. **P < 0.01 (one way ANOVA). n = 6 independent biological replicate experiments; (**B**) AUC was calculated (n = 6), **P < 0.01, two-tailed Student t-test. (**C**) Hepatic p-AMPKa and AMPKa were analyzed by immunoblotting and quantified as relative optical density (**D**). **P < 0.01, two-tailed Student t-test. n = 6 independent biological replicate experiments.

To assess the effect of shizukaol F on gluconeogenesis *in vivo*, we performed a pyruvate tolerance test (PTT), as administration of the gluconeogenic substrate pyruvate increases blood glucose levels by promoting gluconeogenesis in the liver. As described above, mice were pre-treated with 75 mg/kg shizukaol F by gavage, and then were administrated of pyruvate. As shown in Fig. 4A,B, shizukaol F significantly attenuated the blood glucose enhanced by pyruvate compared to control mice. This result indicated that shizukaol F reduced gluconeogenesis *in vivo*. In addition, the phosphorylation of AMPKa in liver isolated from mice treated with shizukaol F was increased about 40%, suggesting shizukaol F activated AMPK *in vivo* (Fig. 4C,D).

### The effect of shizukaol F on glucose metabolism is dependent on the AMPKa phosphorylation activity

**Figure 5:**
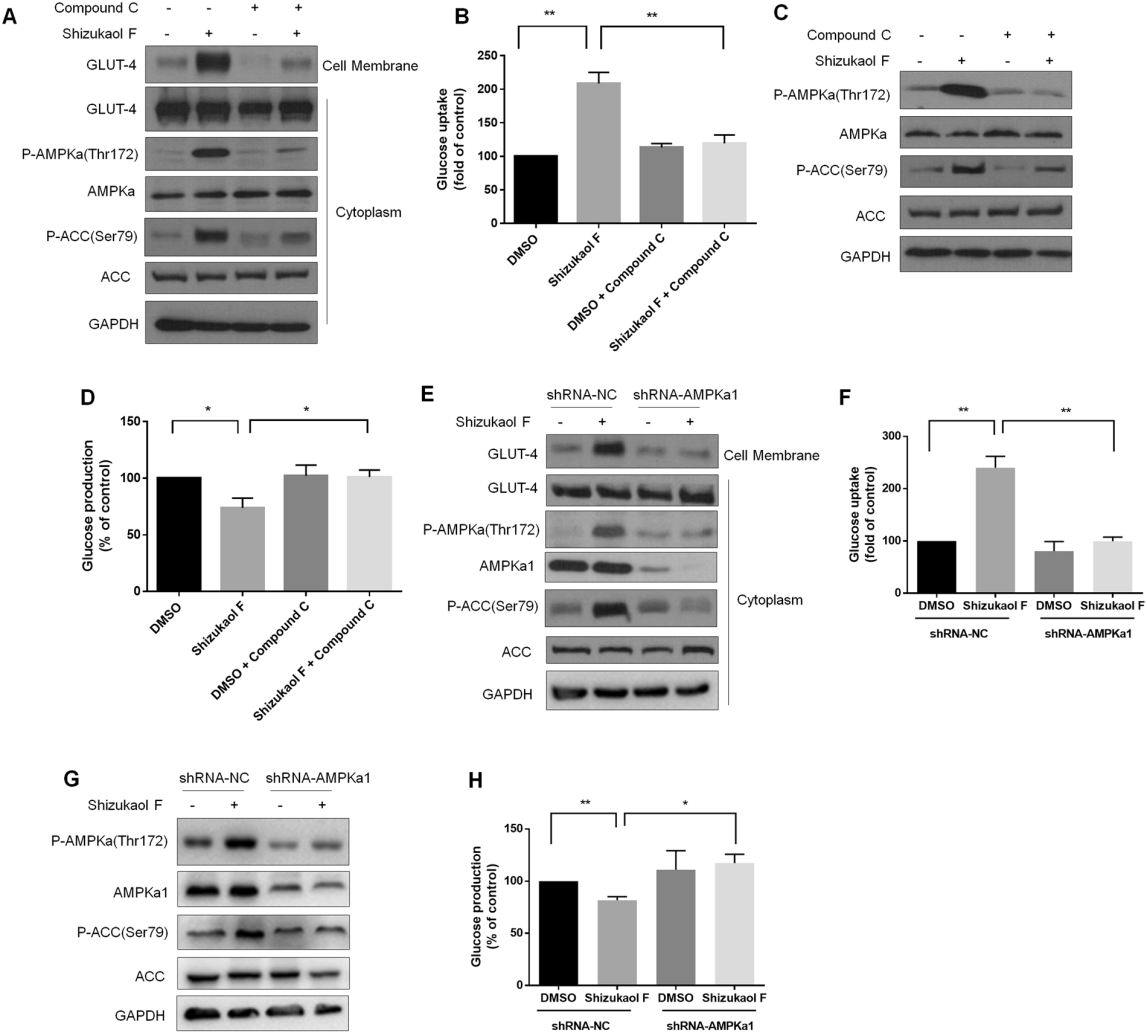
Shizukaol F regulates glucose metabolism via AMPKa phosphorylation activity. C2C12 myotubes were pretreated with 20 μM AMPK inhibitor compound C, and followed by the treatment of 1 μM shizukaol F. Then the immunoblotting analysis of AMPKa phosphorylation and GLUT-4 translocation were measured (**A**); and the determination of glucose uptake (**B**). **P < 0.01 (two-tailed Student t-test). n = 6 independent biological replicate experiments. Primary hepatocytes were pretreated with 10 μM compound C, and followed by the incubation of 1 μM shizukaol F. Then the western blotting (**C**) and gluconeogenesis level were detected (**D**). *P < 0.05; **P < 0.01 (two-tailed Student t-test). n = 6 independent biological replicate experiments. C2C12 cells infected by lenti-virus of shRNA-AMPKa1 were treated with 1 μM shizukaol F for 24 h. AMPKa phosphorylation and GLUT-4 translocation were analyzed by western blotting (**E**). Glucose uptake was measured (**F**). **P < 0.01 (two-tailed Student t-test). n = 3 independent biological replicate experiments. Primary hepatocytes were transfected with shRNA-AMPKa1 lenti-virus or a negative control. Cells were incubation with 1 μM of shizukaol F for 24 h. AMPKa and ACC phosphorylation were analyzed (**G**), and gluconeogenesis level was measured (**H**). *P < 0.05; **P < 0.01 (two-tailed Student t-test). n = 3 independent biological replicate experiments.

To further confirm the relationship between AMPKa phosphorylation and glucose metabolism in response to treatment with shizukaol F, we inhibited AMPKa phosphorylation with the chemical inhibitor compound C^25^. C2C12 cells were pre-treated with 20 μM compound C and then treated with 1 μM shizukaol F. Treatment of the C2C12 cells with compound C significantly inhibited the AMPKa phosphorylation stimulated by shizukaol F (Figs 5A and S4A). In addition, the translocation of GLUT-4 to cell membrane along with the up-regulation of the glucose uptake was blocked by compound C (Figs 5A,B and S4B). Next, the primary hepatic cells were pre-incubated with 10 μM compound C and then treated with 1 μM shizukaol F. As predicted, treatment of the primary hepatic cells with compound C inhibited the phosphorylation of AMPKa induced by shizukaol F (Figs 5C and S4C). Compound C also increased the expression of PEPCK and G6Pase that were suppressed by shizukaol F (Fig. S4D and E). Importantly, the down-regulation of the gluconeogenesis in primary hepatic cells induced by shizukaol F was also blocked by AMPK inhibitor (Fig. 5D).

In addition, we inhibited AMPK activity using a shRNA approach. C2C12 cells were infected with lenti-virus to knockdown AMPKa1 which is a key component of AMPK (Fig. 5E) and then treated the cells with shizukaol F (see Materials and Methods). As expected, the down-regulation of AMPKa1 expression mediated by the shRNA-AMPKa1 resulted in a significant reduction in the levels of phosphorylated AMPKa (Thr 172) and GLUT-4 translocation induced by drug treatment (Figs 5E and S5A,B). Furthermore, knockdown of AMPKa1 significantly reversed the shizukaol F induced glucose uptake (Fig. 5F). Meanwhile, primary hepatocytes were infected with lenti-virus of shRNA-AMPKa1 and then treated with 1 μM shizukaol F. As shown in Figs 5G and S5A, knockdown of AMPKa1 inhibited the phosphorylation of AMPKa (Thr 172) induced by shizukaol F. As a result, knockdown of AMPKa1 stimulated the expression of PEPCK and G6Pase that were inhibited by shizukaol F (Fig. S5D). Importantly, the down-regulation of the gluconeogenesis in hepatocytes induced by shizukaol F was blocked by shRNA-AMPKa1 (Fig. 5H). Taken together, these results strongly supported the conclusion that shizukaol F modulates the glucose metabolism in an AMPKa phosphorylation dependent manner.

### Shizukaol F activates AMPK by inhibiting respiratory complex I

**Figure 6:**
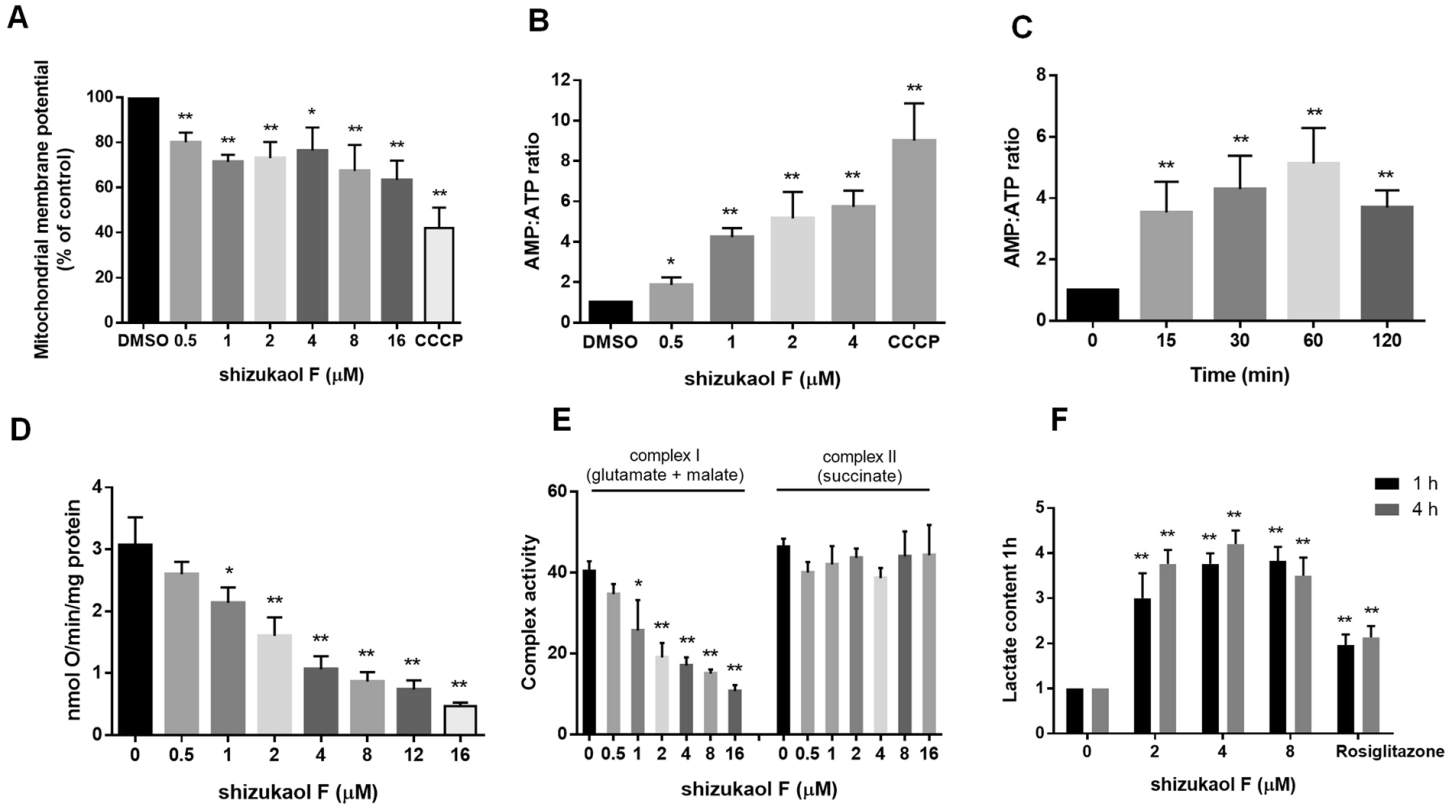
Effect of shizukaol F on the energy status and respiration. (**A**) Dose-dependent depolarization of mitochondrial membrane potential in C2C12 cells for 10 min of shizukaol F treatment at the indicated concentrations. 10 μM CCCP was set as positive control. **P < 0.01 (one way ANOVA). n = 3 independent biological replicate experiments. (**B**) Dose-dependent increase of the AMP/ATP ratio in myotubes exposed to shizukaol F at the indicated concentrations for 2 h. *P < 0.05; **P < 0.01 (one way ANOVA). n = 3 independent biological replicate experiments. (**C**) Time-dependent stimulation of the AMP/ATP ratio in myotubes by 1 μM shizukaol F. **P < 0.01 (one way ANOVA). n = 3 independent biological replicate experiments. (**D**) Inhibition of the respiration of intact C2C12 cells by shizukaol F treatment at the indicated concentrations (n = 3). *P < 0.05; **P < 0.01 (one way ANOVA). (**E**) Effect of shizukaol F on the respiration of isolated mitochondria from mice liver (n = 3). *P < 0.05; **P < 0.05 (one way ANOVA). (**F**) Lactate concentrations of C2C12 cells treated with shizukaol F at indicated conditions were measured. Rosiglitazone (50 μM) was set as a positive control. *P < 0.05; **P < 0.01 (two way ANOVA). n = 3 independent biological replicate experiments.

Several studies have shown that AMPK activating compounds such as metformin and AICAR influence mitochondrial function^10, 26^. We next examined the effect of shizukaol F on mitochondrial membrane potential (Δψm) and energy status. Using a fluorescence detection assay, we first confirmed that shizukaol F depolarized the mitochondrial membrane potential of C2C12 cells in a dose dependent manner (Fig. 6A), though the mitochondrial dysfunction induced by shizukaol F incubation was not as strong as the mitochondrial uncoupling compound CCCP (Fig. 6A). In addition, the inhibition of mitochondrial membrane potential (Δψm) by shizukaol F also led to an increase in the AMP/ATP ratio. As shown in Fig. 6B,C, the increased AMP/ATP ratio in C2C12 cells treated with shizukaol F was detected by HPLC. CCCP and metformin were used as positive control (Figs 6B and S6A). Taken together these results suggested that shizukaol F may activate AMPK through the induction of mitochondria dysfunction, especially energy depletion.

To determine whether the change in nucleotide ratio was due to an impact on cellular respiration, as in case of another AMPK activator such as metformin and AICAR^10, 27^, we examined oxygen consumption in C2C12 cells in the presence of shizukaol F. Treatment with shizukaol F resulted in a dose dependent inhibition of aerobic respiration in C2C12 myotubes (Fig. 6D). Furthermore, the effects of shizukaol F on ADP stimulated respiration in the presence of complex I (glutamate + malate) or complex II (succinate) substrates were measured in isolated mouse liver mitochondria, Rosiglitazone was set as positive control (Fig. S6B,C). Shizukaol F produced a dose dependent inhibition of oxygen consumption with complex I linked substrate, but not with complex II linked respiration (Fig. 6E). Furthermore, shizukaol F increased lactate production in C2C12 myotubes, which is a marker of cellular anaerobic respiration, as the reduction of aerobic respiration may lead to compensated elevation of anaerobic respiration (Fig. 6F). All these findings suggested that shizukaol F modulates AMPKa phosphorylation activity by inhibiting mitochondrial respiratory complex I, which in turn suppresses aerobic respiration and up-regulates anaerobic respiration to meet the energy requirement.

## Discussion

As a natural product from *Chloranthus japonicus*, shizukaol F has been demonstrated to induce various biological activities, including anti-inflammation and anti-HIV activity^20, 22^. But there were no studies showing that shizukaol F has metabolic activities. In the previous study, a natural compound isolated from *Chloranthus japonicus*, shizukaol D was reported to activate AMPK and reduces triglyceride and cholesterol levels in HepG2 cells^17^. To assess the effect of shizukaol F on AMPKa phosphorylation (Thr 172), we first identified that shizukaol F also activates AMPK in a dose-dependent manner. We also showed that shizukaol F modulates glucose metabolism in skeletal myotubes, primary hepatocytes, and in C57BL/6 J mice via AMPK activity. In addition, we found that shizukaol F activated AMPK by inhibiting mitochondria respiratory complex I.

Glucose transport in skeletal muscle is the major component of whole body glucose uptake and plays a key role in maintaining glucose homeostasis^28^. Here we have shown that shizukaol F significantly stimulated glucose uptake in differentiated skeletal myotubes (Fig. 3B). The stimulation of glucose uptake by shizukaol F may be a result of GLUT-4 translocation from cytoplasm to membrane, a downstream effect of AMPK (Fig. 3A). Rosiglitazone was set as positive control since it was used to regulate glucose uptake with the effect of AMPK activation^14^. Meanwhile, the protein of GLUT-4 in cytoplasm was not changed which means shizukaol F increased cellular total GLUT-4 expression. This result was consistent with previously report that AMPK regulates GLUT-4 transcription by phosphorylating histone deacetylase 5 (HDAC5)^29^. In agreement with our studies in C2C12 myotubes, shizukaol F also activated AMPK in primary hepatic cells isolated from C57BL/6 J mouse and reduced PEPCK and G6Pase expression (Fig. 3C,D), suggesting that the decreased expression of gluconeogenic genes may also contribute to the glucose lowering effect of shizukaol F (Fig. 3E). In addition, acute effect of shizukaol F on gluconeogenesis and hepatic AMPKa phosphorylation in C57BL/6 J mice were measured (Fig. 4). Taken together, our results indicated that shizukaol F improves overall glucose metabolism, an effect likely to be mediated by the activation of AMPK.

To confirm the significance of AMPKa phosphorylation in the activity of shizukaol F, we inhibited AMPKa phosphorylation by using AMPK inhibitor compound C and shRNA lenti-virus^25^. As shown in Fig. 5, compound C and shRNA-AMPKa1 caused a remarkable inhibition of AMPK pathway, and decreased shizukaol F mediated phosphorylation of AMPKa. In addition, inhibition of AMPKa phosphorylation suppressed glucose uptake in myotubes stimulated by shizukaol F, and rescued gluconeogenesis ability in primary hepatocytes caused by shizukaol F. These finding suggested that the modulation of glucose metabolism by shizukaol F was dependent on AMPK activity.

AMPK is a sensor of whole body energy homeostasis and is activated directly as a result of energy depletion^30^. Metformin and rosiglitazone are well known to increase the ratio of AMP: ATP, which in turn leads the activation of AMPK^12^. Here, we found that the treatment of shizukaol F inhibited mitochondrial membrane potential and cellular respiration. As a result, the cellular AMP/ATP ratio was also increased by shizukaol F (Fig. 6). As we see, 4 mM metformin increased about 4 folds of AMP/ATP ratio (Fig. S6A), which was consistent with previously report^31^. In this story, 1 μM shizukaol F increased about 4 folds of AMP/ATP ratio, suggesting a much lower dosage of shizukaol F can be used in future treatment. We further investigated whether shizukaol F inhibited the respiratory complex. Surprisingly, we found that shizukaol F inhibited mitochondrial respiration complex I (glutamate and malate) but not complex II (succinate) (Fig. 6E). This finding suggested that shizukaol F modulates AMPK activity by inhibiting mitochondrial respiratory complex I, which therefore led to an elevated AMP/ATP ratio.

In conclusion, our studies demonstrated the beneficial effects of a natural product, shizukaol F, as a potent activator of AMPK regulating glucose homeostasis both *in vitro* and *in vivo*. Shizukaol F caused inhibition of mitochondrial respiratory complex I, thus leading the activation of AMPK and subsequent beneficial metabolic outcomes, including enhanced glucose uptake in skeletal muscle cells and suppression of hepatic gluconeogenesis. These results highlight the value of shizukaol F as a potential compound for the treatment of metabolic diseases.

## Materials and Methods

### Materials

1, 5-aminoimidazole-4-carboxamide-1-D-ribofurano-side (AICAR); 1-dimethylbiguanide (metformin); 5,5′, 6,6′-tetrachloro-1; 1′, 3,3′-tetraethyl-imidacarbocyanine iodide (JC-1); rosiglitazone; carbonyl cyanide m-chlorophenylhydrazone (CCCP); adenosine 5′-triphosphate (ATP) disodium salt hydrate; adenosine 5′-diphosphate sodium salt (ADP); 8-bromoadenosine 3′,5′-cyclic monophosphate (AMP); Glucose (GO) assay kits were purchase from Sigma Aldrich (St. Louis, MO, USA). 6-(4-(2-piperidin-1-ylethoxy) phenyl)-3-pyridin-4-ylpyrazolo (1, 5-a) pyrimidine (compound C) was purchased from Merck Millipore (Darmstadt, Germany). Antibodies against AMPKa, AMPKa1, phosphor-AMPKa (Thr 172), GLUT-4, ACC, phosphor-ACC (Ser79), GAPDH were purchase from Cell Signaling Technology (Beverly, MA, USA). MyoD and myogenin antibodies were got from abcam (Cambridge, MA, USA). The MTT cell proliferation and cytotoxicity assay kits were obtained from the Beyotime Institute of Biotechnology (Jiangsu, China). The lactate assay kit was obtained from the Nanjing Jiancheng Biongineering Institute (JiangSu, China).

### Animals

C57BL/6 J male mice were purchased from SLAC Laboratory Animals (Shanghai, China). The animals were maintained under a 12 h light-dark cycle with free access to food and water. Animal experiments were approved by the Animal Care and Use Committee, Shanghai Institute of Biochemistry and Cell Biology. Animal experiments were carried out in accordance with the relevant guidelines (IBCB-SPF0034). All efforts had been done to minimize the number of animals and decrease their suffering.

### Shizukaol F preparation and purification

*Chloranthus japonicus* is widely distributed in eastern Asia, including mainland China and Japan, and is not an endangered or protected species in China. The plant materials in our experiment were purchased from the Chinese medicinal material market in Panshi, Jilin Province, China. The air-dried and powdered *Chloranthus japonicus* plants (10 kg) were extracted and purified (at least 98%) as previously described^21, 22^.

### Cell Culture

C2C12 cells (American Type Culture Collection, VA) were cultured in Dulbecco’s modified Eagle Medium (DMEM) supplemented with 10% FBS (GIBCO) and 100 units/ml penicillin and streptomycin at 37 °C in 5% CO_2_. For differentiation, cells were washed with PBS and incubated with 2% horse serum for 6 days. Primary hepatocytes were isolated from 12 week male C57BL/6 J mice as previously reported^32^. Cells were cultured in DMEM with 10% FBS.

### Determination of glucose uptake and isolation of plasma membrane

Glucose uptake activity was measured by 2-deoxy-D-glucose uptake as described previously^33^. C2C12 myotubes cultured in 35 mm dishes were serum starved with DMEM for 4 h, washed three times with warm KRH buffer (25 mM HEPES, pH 7.4, 120 mM NaCl, 5 mM KCl, 1.2 mM MgSO_4_, 1.3 mM CaCl_2_, 1.3 mM KH2PO_4_) and then incubated in KRH buffer at 37 °C. Subsequently, the cells were stimulated with 100 nM insulin for 30 min and 2-deoxy-D-[1-^14^C] glucose was added during the last 5 min at a final concentration of 50 μM with 0.2 μCi/ml. Glucose uptake was terminated by ice cold phosphate buffered saline (PBS) washing. The cells were lysed with 1% SDS and subjected to liquid scintillation counting.

The plasma membrane isolation was described previously^33^. Cells were washed with cold HES buffer (20 mM HEPES, Ph7.4, 1 mM EDTA, 0.25 M sucrose) three times and homogenized with HES buffer plus protease inhibitor cocktails. The cell lysate was centrifuged 16000 g for 20 min at 4 °C and the membrane pellet was re-suspended in HES buffer. The plasma membrane was isolated by re-suspend onto a 1.12 M sucrose cushion and centrifuged at 100000 g for 60 min. The membrane fraction on the density interface was collected, diluted with 20 mM HEPES, pH 7.4 and 1 mM EDTA solution and pelleted by centrifugation.

### Mammalian lenti-viral shRNAs

Lenti-viral short hairpin RNA (shRNA) expression vectors and virus were purchased from GenePharma (Suzhou, China). To generate the lenti-viruses, shRNA plasmids were co-transfected into HEK293T cells along with envelope (VSVG) and packaging (Delta 8.9) plasmids using lipofectamine 2000 (Invitrogen). The viral supernatants were harvested and filtered after two days transfection. C2C12 cells were infected in the presence of a serum-containing medium supplemented with 8 μg/ml polybrene. Following infection for 48 hours, cells were selected with 2.0 μg/ml puromycin (Sigma). Knockdown efficiencies were examined by western blot.

### Western blotting analysis

Cells were harvested and lysed in SDS buffer (1% SDS, 10 Mm HEPES, Ph 7.0, 2 mM MgCl_2_, universal nuclease 20 U/ml). Total cellular protein concentration was measured by the BCA method. Equal amounts of the protein samples (25 μg) were subjected to SDS-PAGE and transferred to polyvinylidene difluoride membranes (Millipore, Bedford, MA, USA). The membranes were then blotted with primary antibodies over night at 4 °C (dilution ratio is 1:1000), which is followed by incubation with the secondary antibody (1:10000). The proteins were developed using enhanced chemiluminescence exposed on autoradiograph film or detected using a FUJIFILM western blotting detection system (LAS-4000, FUJIFILM). The p-AMPKa/AMPKa ratio was quantified by densitometry (FUJIFILM Multi Gauge Version 3.0).

### Quantitative real-time PCR (qRT-PCR) assay

Total RNA was isolated from cells using TriZOL reagent (Invitrogen). One microgram total RNA was reverse transcribed using SuperScript II reverse transcriptase kit (Invitrogen). qPCR was performed using the Power SYBR Green Mix (Applied Biosystems) and normalized to GAPDH expression. The following primers were used: GAPDH sense 5′-ACAACTTTGGTATCGTGGAAGG-3′, GAPDH antisense 5′-GCCATCACGCCACAGTTTC-3′. PEPCK sense 5′-CTGCATAACGGTCTGGACTTC-3′, PEPCK antisense 5′-GCCTTCCACGAACTTCCTCAC-3′, G6Pase sense 5′-CGACTCGCTATCTCCAAGTGA-3′, G6Pase antisense 5′-GGGCGTTGTCCAAACAGAAT-3′.

### Mitochondrial membrane potential assay

The mitochondrial membrane potential assay was performed as described previously^16, 17^. C2C12 cells were seeded into black 96-well optical-bottom plates (Corning, Costar). The cells were incubated with shizukaol F or CCCP at 37 °C for 10 min, and then 100 μl of fresh medium containing 0.2 μg JC-1 was added to each well. The plates were incubated at 37 °C for another 20 min, followed by washing three times with 200 μl of Krebs-Ringer phosphate HEPES buffer. The fluorescence was measured at 530 nm/580 nm (red) excitation and emission (ex/em) wavelengths and then at 485 nm/530 nm (green) ex/em wavelengths. The ratio of red to green fluorescence reflects the mitochondrial membrane potential (Δψm).

### Adenine nucleotide extraction and measurement

C2C12 cells were cultured in 60-mm dishes with shizukaol F or CCCP for the indicated time. The samples for cellular adenine nucleotide measurement were prepared and analyzed as described previously^17^. The cells were washed with PBS buffer (140 mM NaCl, 2.7 mM KCl, 10 mM Na_2_ HPO_4_, 1.8 mM KH_2_PO_4_) and trypsinized. Next, the cells were suspended in 4% (vol/vol) perchloric acid and incubated on ice for 30 min. The pH of the lysates was adjusted to between 6 and 8 with 2 mol/l KOH and 0.3 mol/l MOPS. The precipitated salt was separated from the liquid phase by centrifugation at 13000 rpm at 4 °C for 15 min. Adenine nucleotide measurements were conducted by HPLC (Agilent 1200 series) using a C18 column. The HPLC buffer contained 20 mM KH_2_PO_4_ and 3.5 mM K_2_HPO_4_ 3H_2_O at pH 6.1 with the flow rate of 1.0 mL/min. The order of eluted nucleotides was ATP, ADP, and AMP. Standards (7.5 μM ATP, ADP, and AMP in ddH_2_O) were used to quantify the samples.

### Measurement of respiration in C2C12 cells and isolated Mitochondria

Liver mitochondria were prepared from C57BL/6 J mice. Isolated liver was chopped, homogenized (Polytron Homogeniser, Switzerland) and centrifuged (10 g) as described in a previous study^34^. Mitochondria were re-suspended in respiration buffer at a concentration of 60 mg/ml. Respiration measurements in C2C12 cells and isolated mitochondria were performed in a 782 2 channel oxygen system (Strathkelvin Instruments, UK). For mitochondria, the respiration medium contained 225 mM mannitol, 75 mM sucrose, 10 mM Tris-HCl, 10 mM KH_2_PO_4_, 10 mM KCl, 0.8 mM MgCl_2_, 0.1 mM EDTA, and 0.3% (wt/vol) fatty acid-free BSA, pH 7.0. The respiration medium used for the C2C12 cells consisted of 25 mM glucose, 1 mM pyruvate, and 2% (wt/vol) BSA in PBS, Ph 7.4.

### Gluconeogenesis in primary cultured mouse hepatocytes

Primary hepatocytes were isolated by collagenase digestion from mice that had been fasted for 24 h as previously described^32^. Cells were washed with PBS and cultured in glucose production buffer consisting of glucose-free DMEM (Ph 7.4), without phenol red, supplemented with 20 mM sodium lactate and 2 mM sodium pyruvate. After incubation with shizukaol F or DMSO, the medium was collected and the glucose concentration was measured with a colorimetric glucose (GO) assay kit (Sigma). The results were normalized to the total protein content.

### Acute shizukaol F administration and pyruvate tolerance test

75 mg/kg shizukaol F or vehicle (0.5% carboxymethyl cellulose, wt/vol) was administrated by gavage to overnight fasting male C57BL/6 J mice (10 week) 2 h prior to intraperitoneal pyruvate challenge (2 g/kg). Blood glucose values were measured at 0, 15, 30, 60, 90 and 120 min after pyruvate loading via blood drops obtained by clipping the tail of the mice using an ACCU-CHEK advantage II glucose monitor (Roche, IN). The animals were killed and livers were isolated immediately and frozen in liquid nitrogen for immunoblotting analysis^16^.

### Determination of lactate content

C2C12 cells were cultured in a 24-well plate and treated with shizukaol F or 50 μM rosiglitazone (as a positive control) in serum-free cell culture medium for 1 or 4 hours. The amount of lactate in the medium was measured using a lactate assay kit (Nanjing Jiancheng Bioengineering Institute, Nanjing, China).

### Statistics

Results were calculated as the mean ± S.D., and statistical analysis was performed using SPSS and GraphPad Prism 6.0. The level of significance for the difference between data sets was assessed using ANOVA test or two-tailed Student t-test. A p-value of <0.05 was considered significant.

## Electronic supplementary material

supplementary information
