# Supplementary material for: Modulation of glucose metabolism by a natural compound from Chloranthus japonicus via activation of AMP-activated protein kinase
Source: Sci Rep. 2017 Apr 10;7:778. doi: 10.1038/s41598-017-00925-y (PMC5429703; doi:10.1038/s41598-017-00925-y)

Modulation of glucose metabolism by a natural compound from *Chloranthus japonicus* via activation of AMP-activated protein kinase

Rongkuan Hu[1, 3], Huan Yan[2], Xiaoyan Fei[5], Haiyang Liu[2*], and Jiarui Wu[1, 3, 4*]

[1]Hefei National Laboratory for Physical Sciences at Microscale and School of Life Sciences, University of Science & Technology of China, Hefei, 230027, China

[2]State Key Laboratory of Phytochemistry and Plant Resources in West China, Kunming Institute of Botany, Chinese Academy of Sciences, Kunming 650201,China

[3]Key Laboratory of Systems Biology, Institute of Biochemistry and Cell Biology, Shanghai Institutes for Biological Sciences, Chinese Academy of Sciences, Shanghai 200031, China

[4]School of Life Science and Technology, ShanghaiTech University. Shanghai 201210, China

[5]Department of Biochemistry, University of Texas Southwestern Medical Center, Dallas, TX 75390, USA

* Corresponding authors:

Haiyang Liu: haiyangliu@mail.kib.ac.cn;

Jiarui Wu: wujr@sibs.ac.cn

Supporting Information:

Figure S1. The survival analysis of shizukaol F treated C2C12 cells and primary hepatocytes. (A) Viability of C2C12 cells treated with shizukaol F at indicated concentrations for different time-points was analyzed by MTT assay. Result was normalized to DMSO group which is set as 100%. Error bars represent s.d. from three independent experiments. (B) Viability of primary liver cells treated with shizukaol F at indicated concentrations for different time-points was analyzed by MTT assay. Result was normalized to that of DMSO treated cells which is set as 100%. Error bars represent s.d. from three independent experiments. *, P<0.05; **, P<0.01 compared to corresponding controls (one way ANOVA).

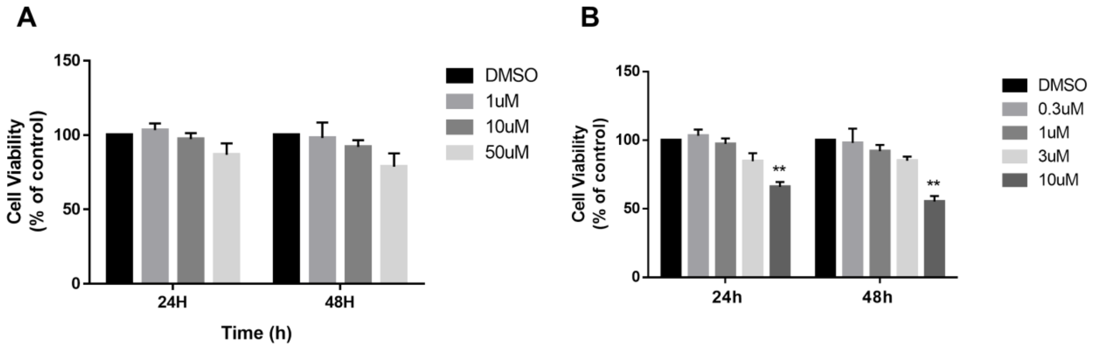

Figure S2. C2C12 differentiation status detected by western. C2C12 cells differentiation was stimulated by 2% horse serum (6 days) and then cells were photographed by fluo-microscopy (A). Two differentiated protein marker, myoD and myogenein, were detected by immunoblotting (B).

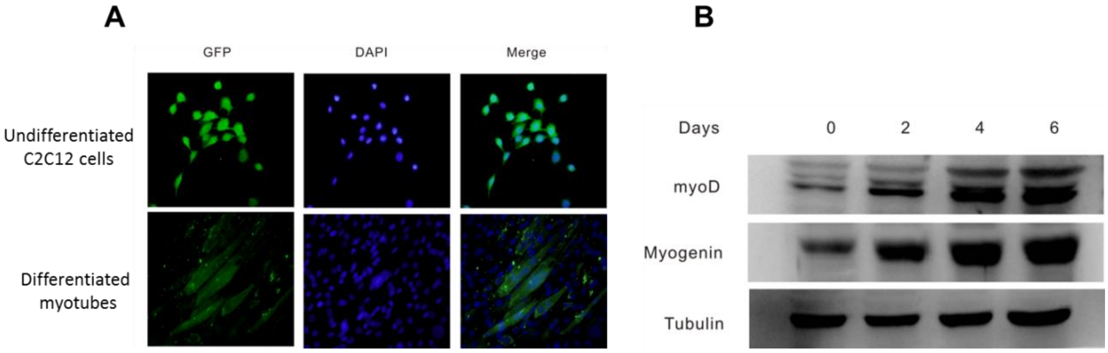

Figure S3. Shizukaol F increases AMPKa phosphorylation in C2C12 cells and primary hepatocyte. C2C12 cells were treated with shizukaol F at indicated concentrations, the phosphorylation levels of AMPKa (A), GLUT-4 translocation (B) and phosphorylated ACC were quantified (C). *, P<0.05; **, P<0.01(one way ANOVA test), n=3 independent biological replicate experiments. (B) The phosphorylation level of AMPKa was quantified after treatment of shizukaol F in primary hepatocytes. **, P<0.01(one way ANOVA test), n=3 independent biological replicate experiments.

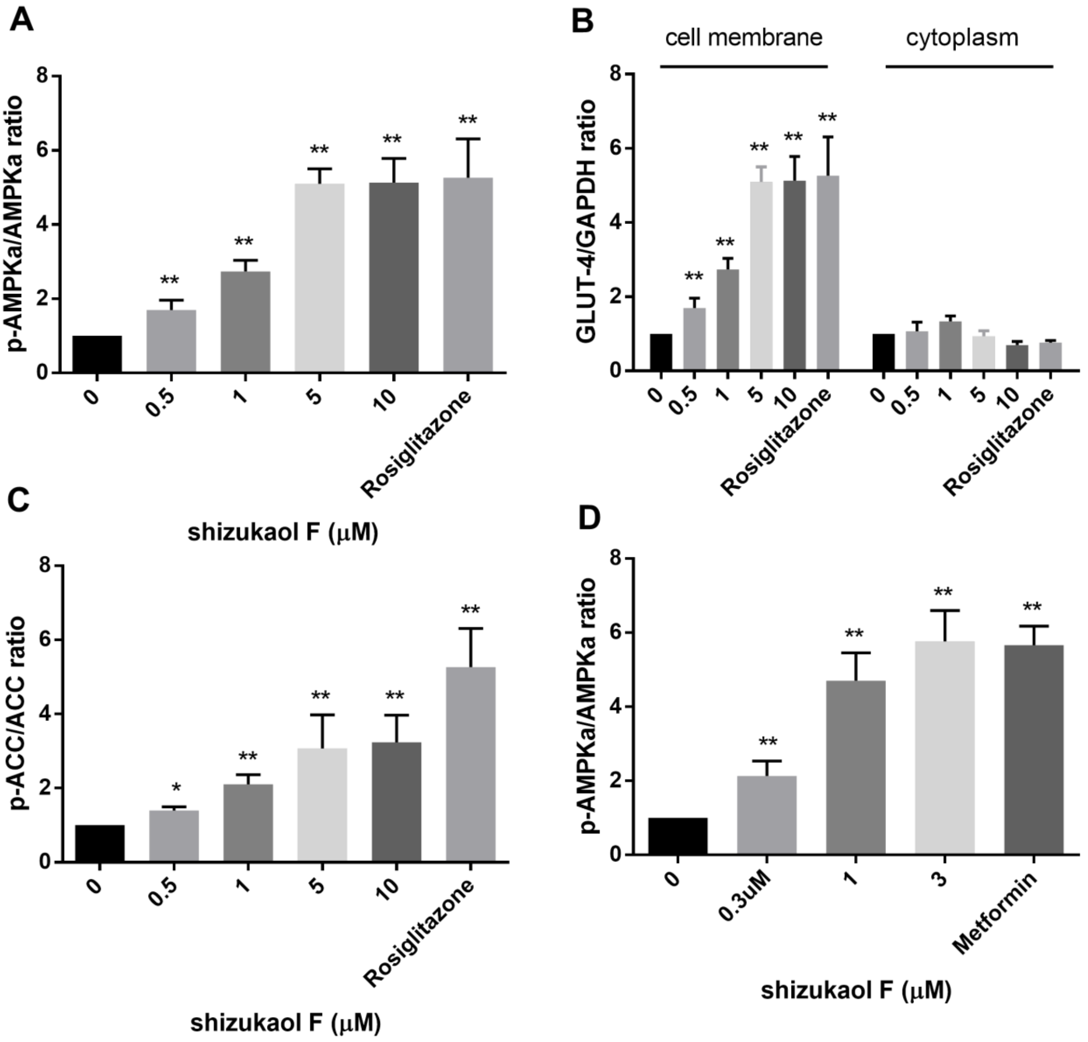

Figure S4. AMPKa phosphorylation activity affects shizukaol F's function. C2C12 myotubes were pretreated with 20 μM AMPK inhibitor compound C, and followed by the treatment of 1 μM shizukaol F, the phosphorylation levels of AMPKa (A) and GLUT-4 translocation (B) were quantified. Primary hepatocytes were pretreated with 10 μM compound C, and followed by the incubation of 1 μM shizukaol F. Then the phosphorylation levels of AMPKa was quantified (C). PEPCK and G6Pase level were detected by q-PCR (D, E). **, P<0.01(two-tailed Student t-test). n ≥ 3 independent biological replicate experiments.

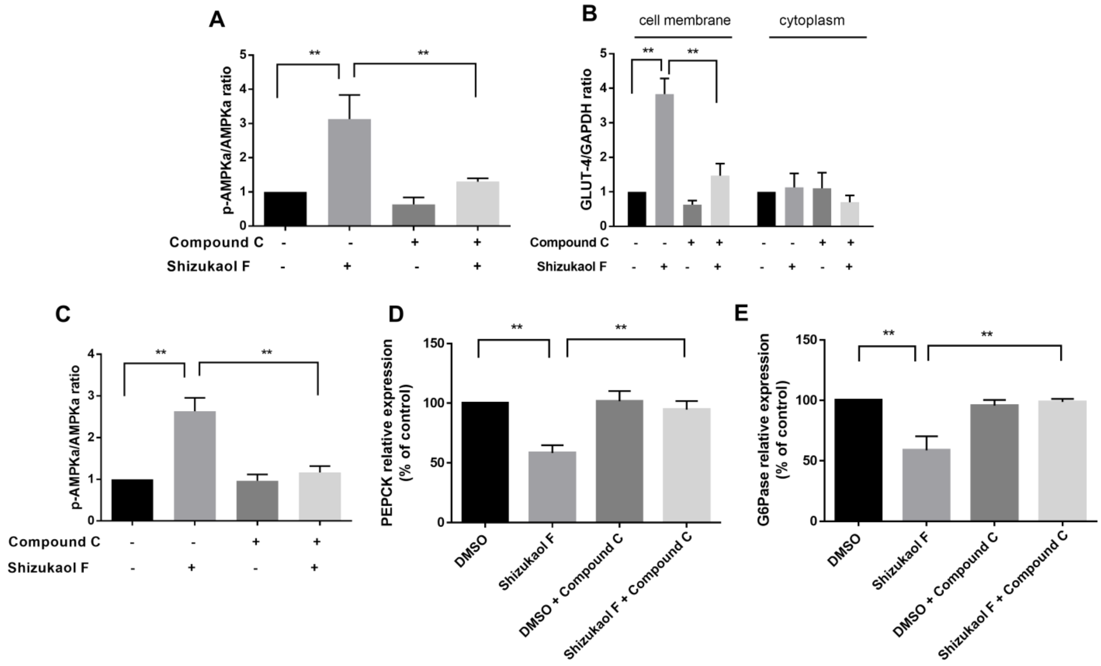

Figure S5. Shizukaol F regulates glucose metabolism via AMPKa phosphorylation activity. C2C12 myotubes infected by lenti-virus of shRNA-AMPKa1 were treated with 1 μM shizukaol F. AMPKa1 relative ratio (A), AMPKa phosphorylation and GLUT-4 translocation were quantified (B). Primary hepatocytes were transfected with shRNA-AMPKa1 lenti-virus or a negative control. Cells were incubation with 1 μM of shizukaol F for 24 h. AMPKa1 relative ratio and AMPKa phosphorylation were calculated (C). PEPCK and G6Pase level were detected by q-PCR (D, E). **, P<0.01(two-tailed Student t-test). n ≥ 3 independent biological replicate experiments.

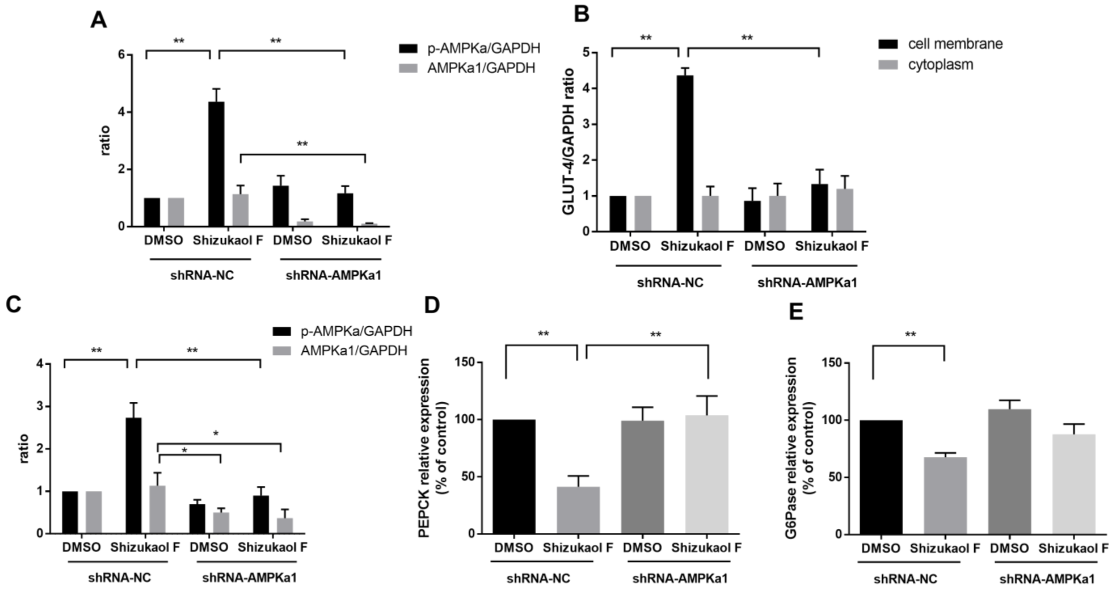

Figure S6. Analysis of respiration in intact C2C12 cells or in mitochondria isolated from C57BL/6J mice. (A) Metformin was set as control in cellular AMP/ATP ratio detection. (B) Rosiglitazone was set as control in cellular respiration analysis. By analyzing the ADP-stimulated respiration in the presence of complex I (glutamate + malate) or complex II (succinate) substrates in isolated mitochondria from HepG2 cells, rosiglitazone (C) was used as specific inhibitor for complex I. **, P<0.01(one way ANOVA), n=3 independent biological replicate experiments.

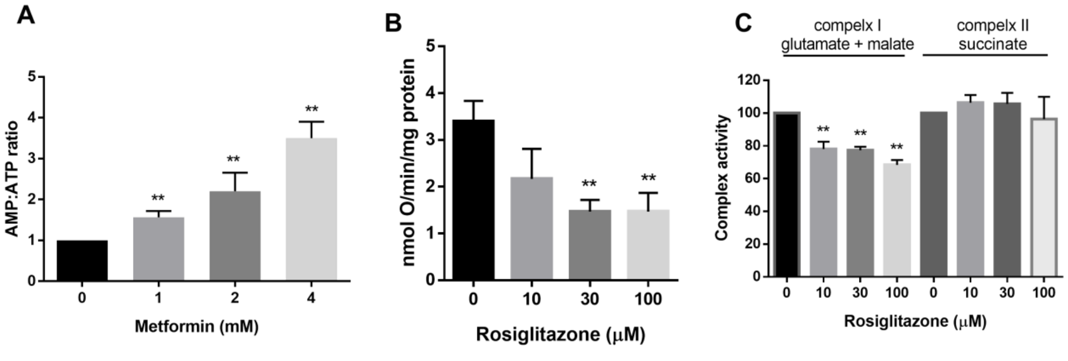

Supplement: Supplementary file 1 — supplementary information [file 41598_2017_925_MOESM1_ESM.pdf]
